# The Protective Effects of *p*-Coumaric Acid on Acute Liver and Kidney Damages Induced by Cisplatin

**DOI:** 10.3390/biomedicines5020018

**Published:** 2017-04-28

**Authors:** Fazile Nur Ekinci Akdemir, Mevlüt Albayrak, Muhammet Çalik, Yasin Bayir, İlhami Gülçin

**Affiliations:** 1 Department of Nutrition and Dietetics, High School of Health, Agri İbrahim Cecen University, Agri 04100, Turkey; 2 Department of Medical Laboratory, Health Services Vocational Training School, Atatürk University, Erzurum 25240, Turkey; mevlutalbayrak@atauni.edu.tr; 3 Department of Pathology, Faculty of Medicine, Atatürk University, Erzurum 25240, Turkey; muhammet.calik@atauni.edu.tr; 4 Department of Biochemistry, Faculty of Pharmacy, Atatürk University, Erzurum 25240, Turkey; ybayir@atauni.edu.tr; 5 Department of Chemistry, Faculty of Science, Atatürk University, Erzurum 25240, Turkey

**Keywords:** hepatotoxicity, nephrotoxicity, *p*-Coumaric acid, antioxidant activity, oxidative stress

## Abstract

In this study, we aimed to investigate the effects of *p*-Coumaric acid (PCA) on cisplatin (CIS)-induced hepatotoxicity and nephrotoxicity in Wistar adult rats for 24 h compared to untreated control groups. In this experiment, 40 Wistar adult rats were utilized and divided randomly into five groups. After 24 h of CIS administration, liver and kidneys were harvested and assessed by H&E staining. Also, markers for oxidative stress and antioxidants were analyzed in theses tissues. Compared to the control group, accumulation of malondialdehyde was increased in groups treated CIS, whereas superoxide dismutase activities and glutathione levels were distinctly diminished in this group. The study’s histopathological findings such as hydropic degeneration, vascular congestion, sinusoidal dilatation in hepatocytes and tubular necrosis in kidneys were in accordance with the results of markers for oxidative stress. PCA may prevent hepatotoxicity and nephrotoxicity by increased antioxidant enzymes and reduced oxidant parameters.

## 1. Introduction

Kidney regulates many necessary functions for body. It removes metabolic products from body [1]. On the other hand, the liver plays an important role in the biotransformation of drugs and toxins, in the fulfilment of many functions such as carbohydrate, fat and protein metabolisms. So, this organ is the main target of drug-induced damage. For a long time, liver and kidney diseases have been increasing worldwide. Metabolic or drug/chemical-induced liver and kidney damage is contributes to these diseases. Overconsumption of a high-fat diet, high-fructose diet [2], high-cholesterol diet [3] as well as alcohol consumption [4], irradiation [5], and some chemicals or drugs including CCl_4_, acetaminophen [6] and cisplatin can also cause liver and kidney damage [7,8].

Cisplatin (CIS) has been effectively utilized as a powerful chemotherapeutic agent against many malignancies, including neck, ovarian, testicular, cervical and bladder cancers [9,10]. In fact, CIS has a crucial role in many cancer treatments. However, it causes a number of undesirable effects involving digestive tract disorders, and increases toxicity in many organs [11]. Although CIS has important side effects on the kidney it is metabolized by the liver [12,13]. Histopathological studies have verified that CIS induces severe liver damage such as degeneration of hepatocytes, and can moderate the dilatation of sinusoids [14,15]. Also, CIS causes kidney damage because it is mainly excreted via the kidneys. The reason for acute renal failure is nephrotoxicity induced by CIS [16]. The negative effects of CIS have been decreased by antioxidant treatment such as ellagic acid, lycopene, vitamin C, macelignan, resveratrol and selenium. Oxidative stress induced by CIS seems to have a major role in hepatotoxicity and nephrotoxicity [14,15,16,17,18,19].

*p*-Coumaric acid (PCA), a phenolic class compound, is obtained from a fraction of plants found in nature [20,21,22,23]. The main sources for phenolic compounds are fruits and beverages like tea, beer and chocolate [24,25,26]. Many researchers have explained the antioxidant mechanism of phenolic compounds, including PCA. So far, many studies have demonstrated the correlations between the consumption of foods and beverages and their protective effects against various diseases [27,28,29]. Recently, interest in food phenolics has increased due to their important roles as antioxidants and free radical scavengers. Also, it has been reported that they protect the human body against various diseases ranging from specific kinds of cancer [30,31,32] to cardiovascular diseases [33].

Up to now, there is no study that directly examines the effects of PCA on hepatotoxicity and nephrotoxicity induced by CIS. In the present paper, we evaluate the protective effect of PCA on tissue damage in CIS-induced hepatotoxicity and nephrotoxicity.

## 2. Material and Methods

### 2.1. Animals and Ethical Decision

Wistar type rats (200 ± 10 g; *n*: 8 per group) were used in our studies. All rats were placed in a room at a fixed temperature (22 ± 1 °C), humidity (55% ± 5%) with equal day/night cycle, with free access to their diet and tap water within their respective cages. The protocol of this experimental research was confirmed by Atatürk University, Local Ethic Committee of the Experimental Animals (2016/3-95). All procedures of our study were done in accordance with the Care and Use of Laboratory Animals Guide.

### 2.2. Drugs and Experimental Models

*p*-Coumaric acid (PCA) and ethanol were obtained from Sigma-Aldrich Chemical Co. CIS was supplied by Koçak Farma Co. (Koçak İstanbul, Turkey, 50 mg/100 mL, intravenous (i.v.)). The rats were randomly separated into five groups including control, control + ethanol, CIS, PCA, and PCA + CIS. Drugs were not applied to the control group. Ethanol (20%, 1 mL, intraperitoneal (i.p.)) was given to the control + ethanol group. PCA was dissolved in 20% ethanol. Later, PCA (100 mg/kg, body weight (b.w.) on three consecutive days before sacrificing; i.p.) was administered to the PCA group. The CIS group was exposed to a single dose of CIS (10 mg/kg b.w., single dose; i.p.) by intraperitoneal injection. PCA and CIS were applied in the PCA + CIS group. All animals were sacrificed under anaesthesia 24 h after CIS administration [34,35,36]. High dose anaesthesia was achieved using thiopental sodium (400 mg/kg b.w.; i.p.). At the end of the experiment, liver and kidney samples were taken and either fixed in 10% formaldehyde for histopathological examinations or stored at −80 °C for subsequent measurement of malondialdehyde (MDA) and glutathione (GSH) levels and superoxide dismutase (SOD) activity.

### 2.3. Biochemical Investigation and Protein Determination

Liver and kidney tissues were kept at −80 °C. Whole liver and kidney samples from each rat were ground in liquid nitrogen using a Tissue Lyser II grinding jar set (Qiagen, Hilden, Germany). All samples were homogenized in 1 mL phosphate buffer solution (0.1 M) homogenate and then centrifuged. GSH [37] and MDA levels [38] and SOD activity [39] from each supernatant sample were measured using by an ELISA reader [40]. The mean values absorbance of the samples was computed. A standard curve was plotted and the equation was acquired from the absorbance of standards. GSH, MDA and SOD proportions were computed appropriating to this equation. The results of the SOD activity, GSH and MDA levels of the tissues were given as U/mg protein, nmol/mg protein, pg/mg protein, and ng/mg protein, respectively. The quantities of proteins in samples were determined according to the Lowry method. Bovine serum albumin was used as the standard commercial protein (Total protein kit-TP0300-1 KT; Sigma Chemical Co. (Munich, Germany)).

### 2.4. Histopathological Examinations

A piece of tissue from each liver and kidney were preserved in formalin solution (10%) for 24 h. Then, the samples were washed using tap water and dilutions of methyl, ethyl and absolute ethyl were applied for dehydration. Specimens were purified in xylene and embedded in paraffin at 56 °C in a hot air oven for 24 h. Paraffin beeswax tissue blocks were prepared for portioning with a thickness of 4 μm by a sledge microtome. The acquired tissue portions were accumulated on glass slides, deparaffinised, and stained by haematoxylin and eosin stain for routine examination. Then, the examination was conducted with a light microscope Carl Zeiss mark Axio imager A2 model. At least five microscopic areas were evaluated to score the specimen. The criteria for liver and kidney injury were hydropic degeneration, vascular congestion, sinusoidal dilatation and tubular necrosis. Each specimen was scored using a scale (−: none, +: mild, ++: moderate, and +++: severe) for each criterion.

### 2.5. Statistical Analyse

All numerical data were examined with a test of one-way analysis of variance. Differences between the groups were evaluated using the Duncan multiple comparison test, which allows inter-comparison of all groups (*p* < 0.05). Our statistical analysis results are given as the mean ± standard error (SEM).

## 3. Results

### 3.1. Markers of Oxidative Stress in Liver and Kidney

**Figure 1:**
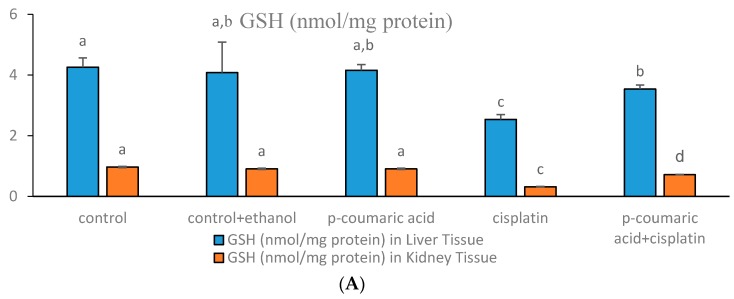

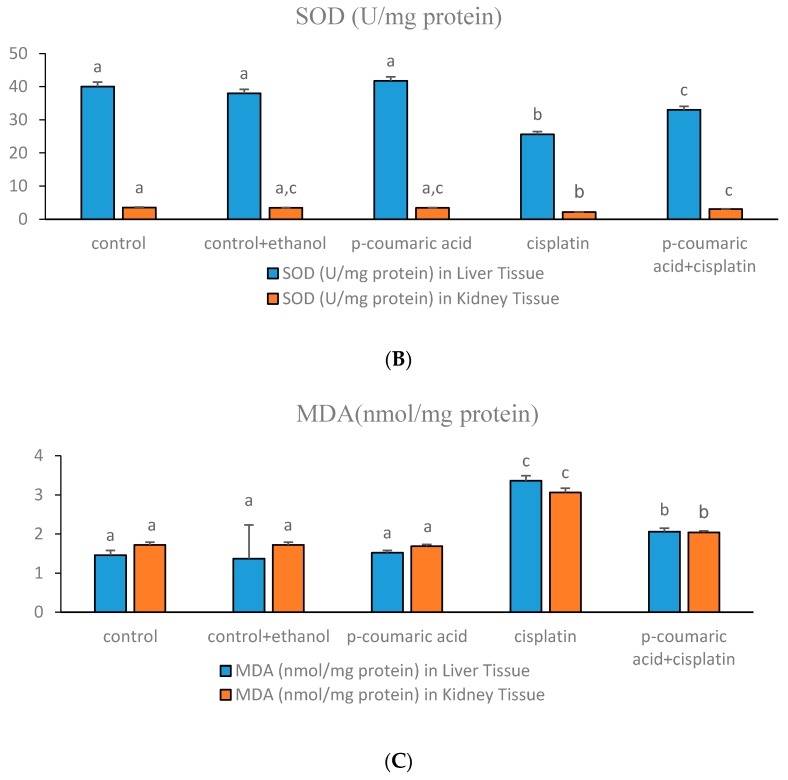
(**A**) Glutathione (GSH) levels in rat liver and kidney tissues. The effects of *p*-coumaric acid (PCA) administration on GSH levels against Cisplatin (CIS)-induced damage in the liver and kidney of rats (mean ± SEM). ^a^ *p* < 0.05 as compared with CIS and PCA + CIS groups. ^c^ *p* < 0.05 as compared with PCA + CIS group and others. ^b^ *p* < 0.05 as compared with the control and CIS groups. ^b^ *p* < 0.05 as compared with cisplatin group and others; (**B**) Superoxide dismutase (SOD) levels in rat liver and kidney tissues. The effects of PCA administration on SOD activity on cisplatin-induced damage in the liver and kidney of rats (mean ± SEM). ^a^ *p* < 0.05 as compared with cisplatin and PCA + CIS groups. ^c^ *p* < 0.05 as compared with the control and CIS groups. ^b^ *p* < 0.05 as compared with the control, control + ethanol, PCA and PCA + CIS groups; (**C**) Malondialdehyde (MDA) levels in rat liver and kidney tissues. The effects of PCA administration on MDA levels in CIS-induced damage in the liver and kidney of rats (mean ± SEM). ^a^ *p* < 0.05 as compared with CIS and PCA + CIS groups, ^c^ *p* < 0.05 as compared with all other groups, ^b^ *p* < 0.05 as compared with the control, control + ethanol, PCA and PCA + CIS groups.

The effects of PCA treatment on oxidative stress parameters like GSH, SOD, and MDA in CIS-induced toxicity in the liver and kidney tissues of experimental animals are shown in Figure 1A–C. The SOD activity in the CIS-treated group was significantly decreased (*p* < 0.05) in the liver and kidney tissues (Figure 1B) compared to the control. The SOD activities significantly increased in the PCA + CIS group. In the control + ethanol and only PCA groups, SOD activities were similar to the control group in both liver and kidney tissues. As can be seen in Figure 1A, the GSH levels in the liver and kidney tissues were extremely low in a high CIS dose-treated group (10 mg/kg b.w.). This difference was found as significant from the control group (*p* < 0.05). GSH levels significantly increased (*p* < 0.05) in the PCA-treated group in liver tissue. Moreover, the GSH level in all the liver groups were higher than the control group. Regarding the influence of PCA on GSH levels in kidney tissue, GSH levels significantly increased depending on treatment with PCA at the 100 mg/kg dose. As can be seen in Figure 1C, MDA levels were diminished by PCA in the liver. Significant effects were observed in comparison with the CIS-treated group (*p* < 0.05). Kidney MDA levels were significantly suppressed by PCA co-administration (*p* < 0.05) when compared to the effects of CIS treatment. In the control-ethanol group and the group treated only with PCA, MDA levels were in the same direction with those observed in the control group.

### 3.2. Histological Scores and Changes in Liver and Kidney Samples

**Figure 2:**
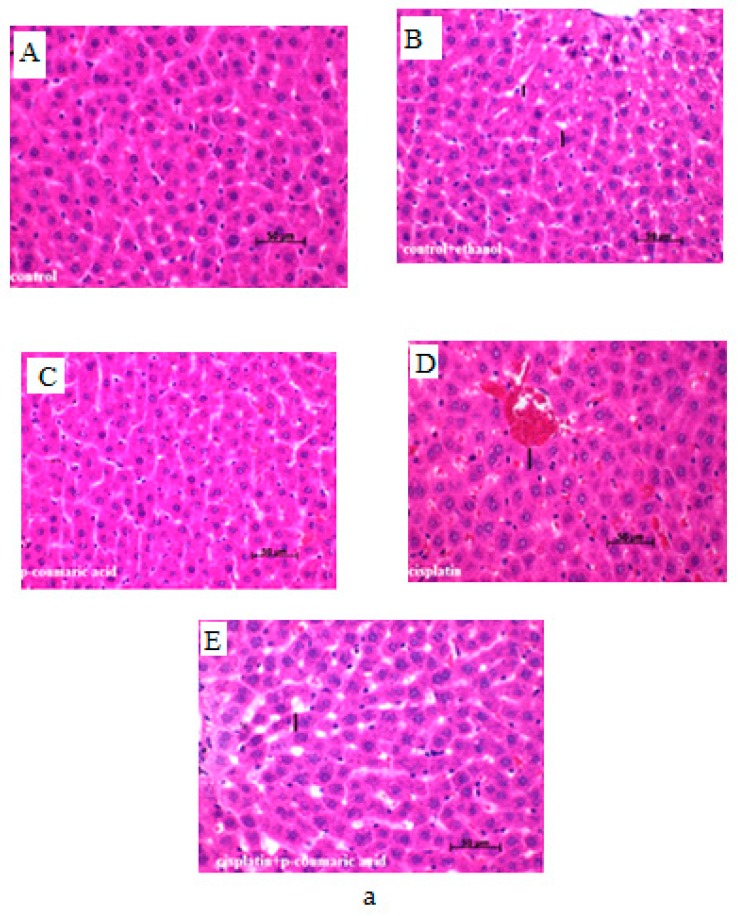

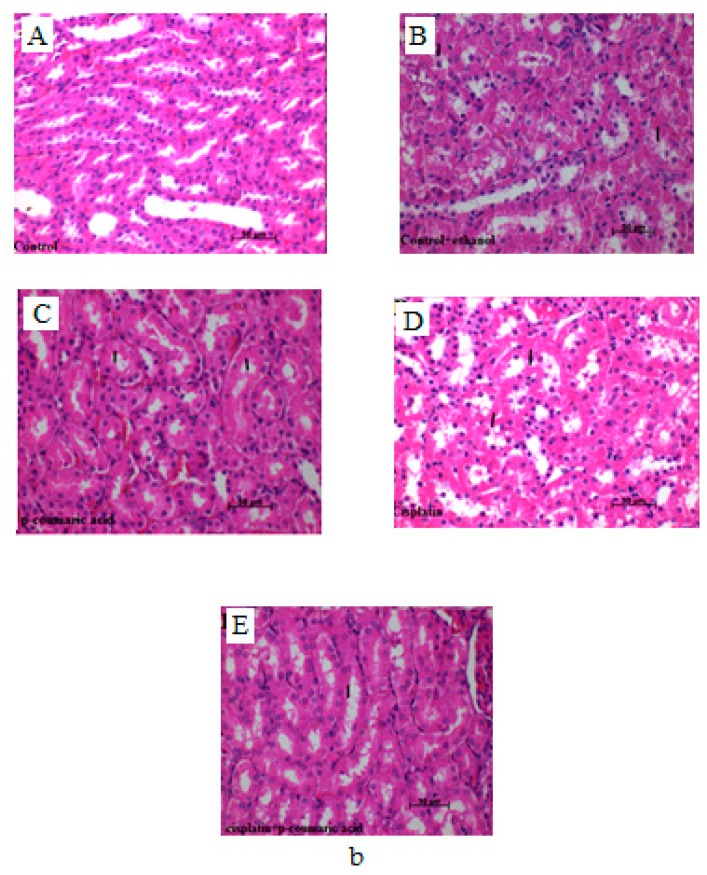
(**a**) Photomicrographs of liver sections from: control; control + ethanol; CIS 10 mg/kg b.w., i.p.; PCA 100 mg/kg b.w. i.p.; and PCA (100 mg/kg b.w.) + CIS (10 mg/kg b.w.) groups. H&E, original magnification 40× or 100×. (**A**) Normal rat liver showing normal hepatocyte without any change in the control group; (**B**) Rat liver showing normal hepatocyte without any significant change in the control + ethanol group; (**C**) PCA-treated rat liver revealing normal hepatocyte without any damage in the PCA group; (**D**) Rats induced with toxicity by CIS revealing damaged hepatocytes, vascular congestion, and sinusoidal dilatation in the CIS group; (**E**) Rats pre-treated with PCA and induced with toxicity by cisplatin revealing near normal liver architecture without vascular congestion or sinusoidal dilatation in the PCA + CIS group; (**b**) Photomicrographs of kidney sections from: control; control + ethanol; CIS (10 mg/kg b.w., i.p.); PCA (100 mg/kg b.w. i.p.); and PCA (100 mg/kg b.w.) + CIS (10 mg/kg b.w.) groups. H&E, original magnification 40 × or 100×. (**A**) Normal rat kidney showing normal kidney cells without any change in the control group; (**B**) Rat kidney showing normal cells without any significant change in the control + ethanol group; (**C**) PCA-treated rat kidney revealing normal cells without any damage in the PCA group; (**D**) Rats induced with toxicity by CIS revealing damaged kidney cells and necrosis of tubular epithelial cells in kidneys in the CIS group; (**E**) Rats pre-treated with PCA and induced with toxicity by cisplatin revealing near normal kidney architecture without necrosis of tubular epithelial cells in the PCA + CIS group.

Hepatic tissues of experimental animals in the control group showed regular cellular structure. Photomicrographs of livers from the CIS-treated animals (Table 1 and Figure 2A) demonstrated signs of injury with noticeable vascular congestion, sinusoidal dilatation and hydropic degenaration in liver tissue. In the PCA + CIS group, improvement of histopathological parameters in liver tissue was detected. In the group treated with CIS 10 mg/kg b.w., crucial changes in the intensity of vascular congestion hydropic degenaration and sinusoidal dilatation were detected when compared to the control, control + ethanol and PCA groups. Some morphological parameters of liver tissue in the PCA + CIS group were attenuated. Results showed that the administration of PCA affected the improvement of the liver histopathological appearance.

Severe pathological changes were observed in the kidney tissues of CIS-treated rats (Table 1). In the control, control + ethanol and PCA groups, normal architecture of the kidney was identified (Figure 2B). In rats only treated with CIS, significant enhancement in tubular cell damage was observed. The parameters of the PCA + CIS group were unable to reduce the significant degeneration and necrosis of tubular epithelial cells.

### 3.3. Discussion

Reactive oxygen species (ROS) are produced from the permeation of electrons into oxygen from different systems in a living organism. As it is known, cellular antioxidant enzymatic and non-enzymatic defence plays a serious role in the mitigation of tissue injury created by free radicals [41,42,43]. These ROS directly act on biological components and produce cellular injury and necrosis in the kidney and other tissues [44,45,46,47]. Actually, the antioxidant system has a primary responsibility in the defence against ROS. Normal injury increases portal and systemic endotoxin levels as well as translocation to the liver, which consequently causes neutrophils recruitment and the further release of ROS [48,49,50]. The formation and eradication of ROS in healthy cells are maintained by a radical scavenging system containing catalase (CAT), superoxide dismutase (SOD), and reduced glutathione (GSH) [51]. Oxidative stress could be a consequence of increased ROS generation and/or decreased antioxidant defence [52,53]. An increased amount of ROS has been reported in toxicity induced by CIS [54,55]. Some antioxidants protected against liver injury induced by high doses of CIS in experimental animals [56]. CIS treatment was found to disrupt the renal antioxidant defence system, as observed in the marked reduction of the activities of all important antioxidant enzymes involving SOD, CAT and GSH-Px both in the renal medulla and cortex [57]. Previous studies have demonstrated increased MDA levels due to CIS-induced nephrotoxicity [58,59]. In this study, the lipid peroxidation was determined by the measurement of MDA levels in rat liver and kidney tissues. MDA levels, which explain the accelerated peroxidation level in the liver and kidney of the CIS group, were higher than in the control group according to our data (Figure 1C). The level of antioxidants may be decreased because of the increase of MDA concentration in CIS-induced tissues. SOD activities and GSH levels were significantly reduced by CIS in liver tissue. The determined reduction in SOD activities and GSH levels might be due to CIS, as suggested earlier [57]. In contrast, a marked increase in the SOD activity was detected when PCA was intraperitoneally given to this group. Some investigators have reported that there is an association between MDA and CIS [60,61]. Our findings confirm the hypothesis that hepatotoxicity and nephrotoxicity result from ROS, which impairs the antioxidant system. Also, our results were compatible with the previous research [57,62]. Additionally, some strategies to treat or prevent liver and kidneys injuries have been studied previously [63].

Histological findings of previous studies demonstrated changes in liver and kidney structure due to CIS treatment. In several studies, sinusoidal dilatation, parenchymal inflammation, vascular congestion in hepatocytes and glomerular and tubular modifications were found to be a result of CIS, and various models that induced hepatotoxicity and nephrotoxicity have been noted [2,50,64,65,66,67,68]. The present histological analysis showed severe degeneration and necrosis of the tubules in kidneys and sinusoidal dilatation, hydropic degeneration, and vascular congestion in hepatocytes of the CIS toxicity group. Meanwhile, the control, control + ethanol and only PCA-treated groups represented regular liver and kidney morphology. The PCA + CIS treatment group demonstrated moderate tubular necrosis in kidneys, indicating the renoprotective ability of PCA. Moderate sinusoidal dilatation, hydropic degeneration, and vascular congestion in hepatocytes was observed in the PCA + CIS group. Also, our histopathological results demonstrated that PCA had positive effects against CIS-induced hepatotoxicity.

The levels of lipid peroxidation products decreased by PCA treatment in rat liver and kidney tissues. This could be due to the inhibitory effect of PCA on lipid peroxidation by virtue of its anti-lipid peroxidation property.

## 4. Conclusions

In the direction of the biochemical and histological findings produced from our study, we conclude that PCA protects the liver and kidney against CIS-induced oxidative damage in the experimental model. Also, PCA inhibits oxidative stress. The observed protective effects of PCA could be attributed to its antioxidant properties. In conclusion, according to the findings of this study, PCA treatment may be beneficial to attenuate oxidative stress in liver and kidney damages induced by cisplatin.

## Figures and Tables

**Table 1:** Effects of *p-*Coumaric acid on morphological parameters of rat liver and kidney tissues after cisplatin treatment.

Groups	Sinusoidal Dilatation in Liver	Vascular Congestion in Liver	Hydropic Degeneration in Liver	Prevalence of Necrosis of Tubular Epithelial Cells in Kidney	Severity of Necrosis of Tubular Epithelial Cells in Kidney
**Control**	− a	− a	− a	− a	− a
**Control + Ethanol**	+ b	+ b	+	+ b	+ b
***p*-Coumaric acid**	− a	+ b	− a	+ b	+ b
**Cisplatin**	+++ d	+++ d	++ c	+++ d	+++ d
***p*-Coumaric acid + Cisplatin**	+ b	++	+ b	++ c	++ c

a: (−), absent; b: (+), mild; c: (++), d: moderate; (+++), marked; Control group; Control + ethanol; Cisplatin 10 mg/kg b.w., i.p.; *p*-Coumaric acid 100 mg/kg b.w. i.p.; *p*-Coumaric acid 100 mg/kg b.w. + cisplatin 10 mg/kg b.w.
